# Ferroptosis Biology and Implication in Cancers

**DOI:** 10.3389/fmolb.2022.892957

**Published:** 2022-04-20

**Authors:** Chi Qu, Yang Peng, Shengchun Liu

**Affiliations:** Department of Endocrine and Breast Surgery, The First Affiliated Hospital of Chongqing Medical University, Chongqing, China

**Keywords:** ferroptosis, iron metabolism, lipid peroxidation, ether lipid, glutathione peroxidase 4, ferroptosis suppressor protein 1, AT-rich interaction domain 1A, chromatin accessibility

## Abstract

Ferroptosis, a novel form of regulated cell death (RCD), has garnered increasing attention in studies on numerous human diseases in the last decade. Emerging evidence has indicated that the pathological process of ferroptosis involves the overloaded production of reactive oxygen species (ROS), followed by aberrant accumulation of lipid peroxidation in an iron-dependent manner, accompanied with an increased uptake of polyunsaturated fatty acids into the cellular membrane, further unfolding an ancient vulnerability in multiple context. The unique nature of ferroptosis differentiates it from other forms of RCD, as it is intricately associated with several biological processes, including the metabolism of iron, amino acids, synthesis of ROS and lipid peroxidation. Accordingly, inducers and inhibitors designed to target the key processes of ferroptosis have been extensively studied. Characterized by its distinct properties as mentioned above and its inducible nature, ferroptosis has been widely implicated in several diseases, and numerous studies have focused on identifying effective therapeutic targets for multiple human diseases, including in cancer, by targeting this process. In the present review, recent studies on the involvement of ferroptosis in several types of cancer are summarized and the findings discussed, highlighting the need for increased contemplation of its involvement in the study of cancer, particularly in the clinical setting. A comprehensive summary of the biological mechanisms underlying ferroptosis, the implications of the multiple inducers of ferroptosis, as well as immunotherapy targeting ferroptosis in different types of cancer is provided in this review to highlight the pathophysiological role of ferroptosis in carcinogenesis, to serve as an aid in future studies on the role of ferroptosis in cancer.

## Introduction

Death, by one of several means, is the eventual fate of cells, and it is physiologically associated with the development and maintenance of the human body, and pathologically associated with the development of numerous diseases (Gao and Jiang, 2018). Certain forms of cell death have been extensively studied and are relatively well understood, and can be inhibited by exposure to treatments that genetically or pharmacologically regulate these processes; these types of cell deaths are therefore termed regulated cell death (RCD) (Galluzzi et al., 2018). The traditional understanding of RCD allows for its stratification into three morphologically distinct types: Apoptosis, autophagy and necrosis. Clinically, chemotherapeutic regimens targeting caspase-dependent apoptosis for the treatment of cancer can result in the acquisition of resistance, thus reducing the efficacy of a chemotherapeutic regimen long term (Gottesman et al., 2002; Angeli et al., 2017). More recently, the discovery of a novel form of RCD highlighted novel opportunities for alternate approaches to treatment of a range of diseases, including cancer (Berghe et al., 2014). Exclusively iron-dependent, ferroptosis is biologically characterized by the overloaded production of reactive oxygen species (ROS) and aberrant accumulation of lipid peroxidation. In addition, cytological changes, including the disappearance of the mitochondrial cristae, outer mitochondrial membrane rupture and the condensation of the mitochondrial membrane, are commonly observed during ferroptosis (Dixon et al., 2012; Stockwell et al., 2017).

Metabolic reprogramming of cancer cells, which results in the changes associated with ferroptosis, were initially described in the 1920s. To prevent the excessive production of ROS, cancer cells preferentially switch from aerobic glycolysis to anaerobic lactate-dependent glycolysis, which in turn increases the requirements of glucose or other energy providing substrates to sustain their proliferative activity, due to the reduced efficiency of anaerobic glycolysis (Goodwin et al., 2014). Correspondingly, the increased metabolic activity enhances the demands on the antioxidant capacity of cancer cells, thus leading the evolution of cells with a reduced sensitivity to conditions of increased oxidative stress (Diebold and Chandel, 2016; Gwangwa et al., 2018). Ferroptosis, unlike other forms of RCD, can trigger the collapse of the antioxidant defense system in cancer cells and potentially presents a more recently evolved but regulatable target for cancer treatment (Dixon et al., 2012).

Studies on ferroptosis address the essential signaling pathways and biomarkers that may serve as therapeutic targets and for prediction of prognosis of certain diseases. Whilst previous studies on this novel form of RCD have highlighted the importance of numerous biological processes, including iron metabolism and the biosynthesis of a range of amino acids and phospholipids (Galluzzi et al., 2018), more recent investigations have provided a broader and clearer insight into the molecular mechanisms underlying ferroptosis. As ferroptosis has been discovered more recently than the other forms of RCD, its value as a targetable mechanism for management of diseases remains to further studied. In the present review, a systematic, comprehensive and up-to-date summary of the studies in this field is provided, with the aim of providing an integrated understanding of ferroptosis biology, both metabolically and molecularly.

## Ferroptosis Biology

**FIGURE 1:**
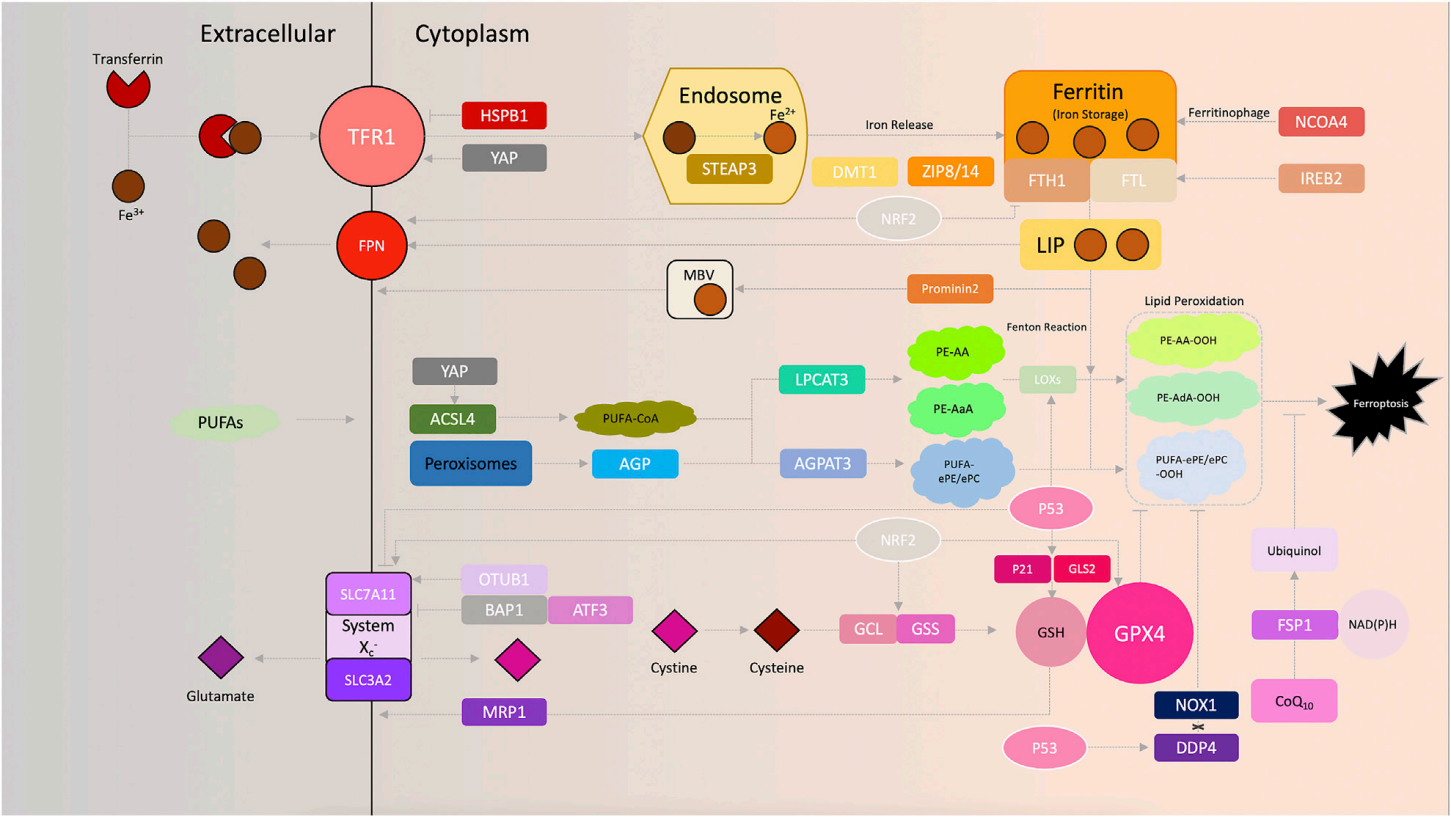
Schematic of ferroptosis signaling.

Recent studies on ferroptosis have improved our understanding of the biological principles and regulatory mechanisms underlying ferroptosis-based oncotherapy. In this section, the key regulators and metabolic mechanisms involved in ferroptosis biology are summarized (Figure 1), with an emphasis on the implications of targeting ferroptotic cell death in cancer treatments. In addition, the metabolic alterations triggered by chromatin remodeling are also discussed and novel perspectives in this area are considered, to highlight potential means of exploiting ferroptosis to improve patient outcomes.

Metabolism of iron. The Fenton reaction, exclusively catalyzed by redox-active iron (Fe^2+^), has been shown to be obligatory for excessive ROS production, which further sensitizes cells to ferroptosis. The delicate nature of intracellular iron transport serves as a key operator of ferroptosis (Dixon and Stockwell, 2014). The addition of the iron chelator deferoxamine, which functionally disables iron utilization, rescues cells from eradicator of Ras and ST (erastin)-induced ferroptosis (Dixon et al., 2012). Complexes of extracellular ferric iron (Fe^3+^) and transferrin can be specifically imported by the transferrin receptor (TFRC) 1 (TFR1) and further shuttled to the endosome, where Fe^3+^ is reduced to ferrous iron (Fe^2+^) by the six-transmembrane epithelial antigen of the prostate 3 (Gao et al., 2015a). Increased iron uptake consequently enhances the susceptibility of cancer cells to oxidative damage and ferroptosis. Ferroptosis-sensitive cells exhibit increased levels of TFR1 (Yang and Stockwell, 2008), whereas inhibition of iron uptake by silencing TFRC, the gene encoding TFR1, results in impaired ferroptosis induced by erastin (Gao et al., 2015a). Overexpression of heat shock protein β-1 (HSPB1) downregulates TFR1 and concurrently inhibits erastin-induced ferroptosis while antagonizing Merlin-Hippo signaling, synchronously activating YAP, leads an upregulated expression of TFR1 and, vice versa, prompts ferroptosis (Sun et al., 2015; Wu et al., 2019). Fe^2+^ is released into the cellular labile iron pool by divalent metal transporter 1 (DMT1) or zinc-iron regulatory protein family 8/14, and is stored in ferritin, a complex of ferritin light chain (FTL) and ferritin heavy chain (FTH1) (Bogdan et al., 2016). Interestingly, our previous study also found that DMT1 was significantly upregulated upon induction of ferroptosis (Yu et al., 2019a). Finally, redundant Fe^2+^ is exported and oxidized to Fe^3+^ by ferroportin to ensure cellular iron homeostasis (Bogdan et al., 2016). Induction of prominin 2, a pentaspanin protein involved in lipid dynamics, facilitates the formation of ferritin-containing multivesicular bodies and exosomes to export iron out of the cells, thereby impeding ferroptosis in breast cancer cells (Brown et al., 2019). Similarly, knockdown of nuclear receptor coactivator 4 decreased the degradation of ferritin, which in-turn reduces the sensitivity to ferroptosis in human pancreatic carcinoma cells (Hou et al., 2016). Additionally, suppression of iron responsive element binding protein 2, a master transcription factor of iron metabolism, increases the iron storage capacity of cancer cells by upregulating FTL and FTH1, thereby inhibiting erastin-induced ferroptosis (Dixon et al., 2012; Gammella et al., 2015). Moreover, RAS signaling was found to be vital for regulating ferroptosis sensitivity by regulating iron metabolism in certain cancer cell lines (Yagoda et al., 2007). As mentioned above, cancer cells have a significantly higher demand for energy and iron, which in turn make them more vulnerable to accumulation of iron and ROS (Hassannia et al., 2019). This biological phenomenon addresses the involvement of iron utilization in carcinogenesis, and reinforces the importance of ferroptosis in future cancer treatments.

Role of amino acids in ferroptosis. Transmembrane transport of amino acids, such as facilitated diffusion, requires specific transporters on the cell membrane. System X_c_^−^, a heterodimeric transporter consists of two functional subunits: Solute carrier family 3 member 2 (SLC3A2) and solute carrier family 7 member 11 (SLC7A11), which are prevalently expressed on phospholipid biolayers (Dixon et al., 2012). With the assistance of system X_c_^−^, glutamate and cystine are exchanged across the plasma membrane and cystine is immediately reduced to cysteine once imported, which is followed by the synthesis of reduced glutathione (GSH). Since glutamate and cystine transport are the most upstream events in ferroptosis, targeting system X_c_^−^ provides a suitable method for induction of ferroptosis (Toyokuni et al., 2017). Erastin was one of the first ferroptosis inducers identified, and functions by directly inhibiting system X_c_^−^ by binding to the mitochondrial voltage-dependent anion channel 2/3 and activating the endoplasmic reticulum (ER) stress response (Dixon et al., 2014). Sulfasalazine, commonly used for treatment of chronic inflammation, was also found to trigger ferroptosis by inhibiting the cystine transporter (Gout et al., 2001; Yu et al., 2019a). In certain cancer cells, sorafenib exerts it toxic effects by suppressing system X_c_^−^ to induce ferroptosis as well (Dixon et al., 2014). The bioactivity of system X_c_^−^ also varies based on the genetic variants of the catalytic subunit, SLC7A11. For example, ubiquitin aldehyde binding 1 (OTUB1) was found to directly interact with and stabilize SLC7A11 in cancer cells. Reducing the cellular SLC7A11 levels by knocking down OTUB1 significantly increased the sensitivity of cells to ferroptosis (Liu et al., 2019). In fibrosarcoma HT-1080 cells, overexpression of activating transcription factor 3 reduced SLC7A11 by directly binding to its promoter region and thereby increasing the sensitivity of cells to erastin-mediated ferroptosis (Wang et al., 2020). Moreover, the tumor suppressor gene, BRCA1-associated protein 1 (BAP1), was found to reduce SLC7A11 expression by decreasing histone 2A ubiquitination occupancy on its promoter in human cancer, which consequently resulted in increased ferroptosis (Zhang et al., 2018).

GSH is on the most abundantly present antioxidants in mammals, where it serves as an electron donor for glutathione peroxidase 4 (GPX4) and thereby reduces lipid hydroperoxide levels. On the basis of glutamate, cysteine and glycine levels, GSH synthesis is strictly dependent on the concentration of substrates and the biological availability of two catalytic enzymes, glutamate-cysteine ligase and GSH synthetase (Meister, 1983). Consistently, inhibitors that prevent GSH biosynthesis can induce ferroptosis. Buthioninesulfoximine (BSO), an inhibitor of γ-glutamyl cysteine synthetase, has been shown to effectively induce ferroptosis in RAS-mutated cells (Yang et al., 2014). By depleting GSH, acetaminophen was found to synchronously induce apoptosis and ferroptosis in specific cell lines as well (Lőrincz et al., 2015). Moreover, a recent study demonstrated that most CD8^+^ memory T cells physiologically possess upregulated levels of cytosolic phosphoenolpyruvate carboxykinase, which consistently ensures high levels of GSH and consequently quenches ROS production to favor cell survival, and GSH has been implicated in the molecular mechanism underlying this protective effect in cancer immunotherapy (Ma et al., 2018). Additionally, cancer cells overexpressing multidrug resistance protein 1, an ATP binding cassette-family transporter, possess increased GSH efflux capacity and thereby exhibit higher susceptibility to ferroptosis (Cao et al., 2019). Together, these observations summarize the potential of targeting glutamate and cystine transport, as well as GSH synthesis as a method of cancer treatment.

Lipid peroxidation. Oxygen (O_2_), which is required for aerobic respiration and is vital for life, receives electrons from NADH, and in the process, is reduced to water (Papa et al., 2012). Simultaneously, a proportion of the electrons may react with oxygen molecules and generate reactive superoxide (O^−2^) in the mitochondria (Sullivan and Chandel, 2014). The high antioxidant capacity present in cells, mediated by certain antioxidant molecules, such as superoxide dismutase, can catalyze O^−2^ into hydrogen peroxide (H_2_O_2_) which is less reactive (Sullivan and Chandel, 2014). In the presence of redox-active divalent iron (Fe^2+^) and H_2_O_2_, the Fenton reaction can be conditionally triggered and hydroxyl radicals (HO·) may thus be generated (Cheng and Li, 2007). These molecules containing a partially reduced oxygen are collectively referred to as ROS, and they can cause aberrant oxidation of DNA/RNA, lipids and proteins, and thus induce cell death (Lin et al., 2018). Indeed, induction of ferrostatin-1 and liproxtatin-1 efficiently inhibit ROS accumulation in multiple types of cancer cells and consequently counteract ferroptosis induced by erastin and RSL3 (Dixon et al., 2012; Friedmann Angeli et al., 2014).

Another hallmark of ferroptosis is the aberrant accumulation of lipid peroxidation (D'Herde and Krysko, 2017). Mechanistically, extensive lipid peroxidation can destabilize and thin the lipid bilayer, which consequently leads to the loss of biomembrane integrity and causes cell death (Agmon et al., 2018). The decomposed lipid peroxides can reactively disrupt and devastate nucleic acids and proteins, further triggering ferroptotic death (Gaschler and Stockwell, 2017). Fueling cells with polyunsaturated fatty acids (PUFA), instead of unsaturated or monounsaturated fatty acids, significantly enhances a cells susceptibility to ferroptosis (Yang et al., 2016); the mechanistic explanation of this phenomena revolves around the increased sensitivity of PUFAs to lipid peroxidation, as they possess highly reactive hydrogen atoms in the methylene bridges (Emerit and Michelson, 1982; Aruoma et al., 2006). Esterification of PUFAs requires the action of Acyl-CoA synthetase long-chain family member 4 (ACSL4), which functionally promotes the synthesis of PUFA-coenzyme A (CoA), particularly arachidonic acid-CoA (AA-CoA) and adrenic acid-CoA (AdA-CoA). In fact, upregulated ACSL4 upon YAP activation prompts ferroptosis (Wu et al., 2019) and ACSL4-knockout cancer cells exhibit significantly decreased levels of AA-CoA and Ada-CoA, and in-turn, induction of ferroptosis by RSL3 is reduced (Doll et al., 2017). Incorporation of PUFAs into the cell membrane contributes to lysophosphatidyl-choline acyltransferase 3, and substrates consisting of AA- and AdA containing phosphatidylethanolamine species are simultaneously generated and presented for lipid peroxidation (Dixon et al., 2015; Hashidate-Yoshida et al., 2015). Interestingly, recent lipidomic profiling revealed that polyunsaturated ether phospholipids (PUFA-ePLs) may serve as another important substrate for lipid peroxidation; peroxisomes were identified to exclusively contribute to ferroptosis by catalyzing the biosynthesis of ether lipids (Zou et al., 2020). In fact, peroxisome components, such as peroxisomal enzymes alkylglycerone phosphate synthase, fatty acyl-CoA reductase 1 and glyceronephosphate O-acyltransferase synergistically promote the biosynthesis of the ether lipids precursor, 1-O-alkyl-glycerol-3-phosphate, which is then transported to the ER (Honsho and Fujiki, 2017). The ER-resident enzyme 1, acylglycerol-3-phosphate O-acyltransferase 3, acylates AGP by adding an ester-linked PUFA, which ACSL4 cooperatively incorporates into the sn-2 position in PUFA-ePL containing PUFA-ether-linked phosphatidylethanolamine, resulting in the generation of PUFA-ether-linked phosphatidylcholine. As a result, sufficient intracellular concentrations of peroxisomes and PUFA-ePL guarantee adequate susceptibility to ferroptosis, and conversely, the biochemical plasticity in attenuating PUFA-ePL in cancer cells allows them to subsequently evade ferroptosis (Zou et al., 2020). Ferroptotic lipid peroxidation specifically requires for divalent iron (Fe^2+^) and hydroxyl radicals (HO·) generated from the Fenton reaction to directly react with and oxidize PUFAs that have been incorporated into membrane phospholipids to trigger ferroptotic cell death. The Fe^2+^ coadjutant lipoxygenase (LOX) can enzymatically facilitate the generation of doubly and triply-oxygenated (15-hydroperoxy)-diacylated PE species as well, which induces ferroptosis (Yang et al., 2016; Kagan et al., 2017). Recently, the LOX family member (ALOX)12 was found to facilitate P53-mediated ferroptosis in an ACSL4-independent manner (Chu et al., 2019), whereas Raf1 kinase inhibitory protein induces activation of ALOX15 and increases the sensitivity of cells to ferroptosis (Anthonymuthu et al., 2018).

Increasing the antioxidant capacity of cells and preventing lipid peroxidation protects cells from ferroptosis. GPX4 was first identified as a mammalian GPX in 1982 (Ursini et al., 1982), and subsequently shown to be a key upstream regulator of ferroptosis in 2014 (Friedmann Angeli et al., 2014; Yang et al., 2014). GPX4 functions to continuously hydrolyze complex lipid hydroperoxides, and GSH serves as an electron donor to constitutively reduce phospholipid hydroperoxides (such as PE-AA-OOH and PE-AdA-OOH) to less oxidative phospholipid alcohols (such as PE-AA-OH and PE-AdA-OH), thus reducing induction of ferroptosis (Ursini et al., 1985). Serving as one of the most important biomarkers of ferroptosis signaling, GPX4 can potentially be targeted pharmacologically or genetically to trigger ferroptotic cell death. Inhibitors, such as RSL3 directly bind and inactivate GPX4 via alkylation of the selenocysteine, and thereby increases lipid peroxidation (Yang et al., 2014). Several ferroptosis-inducing agents (FINs) reduce GPX4 activity without GSH depletion, and the PC-OOH is not reduced, thus inducing ferroptosis (Yang et al., 2014). Moreover, the molecular and metabolic alterations in different cells provides a foundation for understanding the differences in ferroptosis susceptibility, further highlighting other potential exploitable targets for the treatment of cancer. In this regard, studies have used CRISPR-Cas9 screens in GPX4 pharmacologically or genetically attenuated cells, and consequently determined the essential molecules that were potentially responsible for ferroptosis susceptibility, and these studies reinforced the importance of GPX4 in ferroptotic signaling (Havas et al., 2001; Clapier and Cairns, 2009; Gilbert et al., 2014; Yang et al., 2014).

Two recent studies have concurrently determined and described a novel regulator that confers potent protection against ferroptosis induced by GPX4 depletion, termed ferroptosis suppressor protein 1 (FSP1), which was previously referred to as apoptosis-inducing factor mitochondria associated 2 (Bersuker et al., 2019; Doll et al., 2019). In addition to the GPX4-GSH antioxidant capacity, which functions by converting lipid hydroperoxides into non-toxic lipid alcohols, N-terminal myristoylation recruits FSP1, which functions as a lipophilic radical-trapping antioxidant, to reduce coenzyme Q_10_ (CoQ_10_; also known as ubiquinone) to ubiquinol by shuttling reducing equivalents from NAD(P)H into the lipid bilayer, and this consequently inhibits lipid peroxidation and halts ferroptosis. These studies first elaborated the importance of the NADH-FSP1-CoQ_10_ pathway in ferroptosis signaling, and highlighted its potential as a target in oncotherapy. In lung cancer cells, FSP1 expression is significantly upregulated, which allows cells to evade ferroptosis. This result highlights the importance of FSP1 in cell protection and as a potential target for inducing ferroptosis as a treatment for cancer (Bersuker et al., 2019; Doll et al., 2019).

Essential genes comprehensively cross-talking with ferroptosis. P53, conventionally emerged and recognized as a tumor suppressor which exerts its biological essence in regulating cell cycle arrest, apoptosis, senescence (Vousden and Prives, 2009; Kastenhuber and Lowe, 2017), is now identified as the interest of ferroptosis cascade (Liu and Gu, 2022). By binding to the specific p53 response element in the 5’ untranslated region of SLC7A11 and subsequently recruiting chromatin-modifying enzymes, P53 transcriptionally inhibits SLC7A11 and therefore upregulates ferroptosis susceptibility (Brady et al., 2011; Li et al., 2012). ALOX12, which helps to fuel lipid peroxidation by enzymatically facilitating the generation of doubly and triply-oxygenated (15-hydroperoxy)-diacylated PE species during ferroptosis, is also proved a target of P53 (Chu et al., 2019). Moreover, P53-transcriptionally-activated glutaminase 2 (GLS2), promotes cellular antioxidant capacity by increasing GSH production and subsequently regulates ferroptosis (Hu et al., 2010; Bieging et al., 2014; Gao et al., 2015b), while P21, a well-known target of P53, is also claimed to augment cellular GSH (Tarangelo et al., 2018). In mechanical addition, by sequestering dipeptidyl peptidase-4 (DDP4), P53 unleashes NADPH oxidase 1 (NOX1) from plasma membrane and consequently decreases lipid peroxidation (Xie et al., 2017). Further studies aimed at understanding the specific molecular mechanism by which p53 mediates ferroptosis may highlight novel avenues for treatment of cancer, through identification of novel targets.

Nuclear factor erythroid 2-related factor 2 (NRF2), named to initiate antioxidant capacity after being translocated to nucleus, captured its momentous status in ferroptosis signaling due to its comprehensive regulation in iron metabolism and lipid peroxidation (Kerins and Ooi, 2018; Dodson et al., 2019). By transcriptionally upregulating ferroportin1 (FPN1) for iron efflux whilst downregulating ferritin to cut down iron storage, NRF2 impairs ferroptosis fundamentally by means of eliminating iron supply (Harada et al., 2011; Tomiotto-Pellissier et al., 2018). Moreover, important genes involved in glutathione synthesis including glutathione synthetase (GSS), glutamate-cysteine ligase (GCL) and the transporter SLC7A11, are also targeted by NRF2 (Chan and Kwong, 2000; Ishii et al., 2000; Kwak et al., 2002; Sasaki et al., 2002; Yang et al., 2005). Other mechanism by which NRF2 mitigates ferroptosis incorporates upregulation of GPX4, an integral anti-ferroptotic agent as mentioned priorly and impacting on heme oxygenase 1 (HMOX1), to promote ROS production ((Abdalkader et al., 2018; Hassannia et al., 2018; Zou et al., 2019), (Takahashi et al., 2020)). Thus, NRF2 is deemed as a critical factor for ferroptosis due to its uniformed regulation on these vital biological processes and is potentially turned as a target for triggering ferroptosis.

Chromatin accessibility and ROS production. The switching defective/sucrose nonfermenting family (SWI/SNF), equipped with two substantially catalytic ATPase subunits, a helicase-SANT (which contains an N-terminal domain that is bound to actin and a C-terminal bromodomain) induces chromatin remodeling, promotes unfolding and thereby exposes binding sites for transcription factors (Kadoch et al., 2013; Mashtalir et al., 2018). ARID1A, which encodes an important SWI/SNF component, was found to be frequently mutated in several types of cancer, and significantly contributes to tumorigenesis (Kadoch et al., 2013). Recently, ARID1A was shown to be an essential regulator for the maintenance of GSH metabolism in cancer cells. Inhibitors, such as APR-246, PRIMA-1 (which devitalizes cysteine by covalently binding to its residues) and BSO exhibit significantly higher sensitivity in ARID1A-deficient ovarian cancer cells, and thus increase ROS-accumulation-induced cell death. Indeed, cysteine levels are significantly reduced in ARID1A-deficient cells compared with ARID1A-proficient cells, confirming the ability of ARID1A to affect GSH metabolism by regulating the import of cysteine (Ogiwara et al., 2019). Additionally, ARID1A-deficient patient-derived gastric cancer cells are more sensitive to GSH attenuation induced by inhibitors, such as APR-246 and BSO, as well the SLC7A11 inhibitor, erastin (Sasaki et al., 2020). In fact, the expression of SLC7A11 is significantly lower in ARIDA1A-deficient cells. Mechanistically, combined with BRG1 (the catalytic subunit of the SWI/SNF chromatin remodeling complex), ARID1A exposes the transcription start site of SLC7A11, and colocalizes with the transcription factor NRF2, which consequently triggers the transcription of SLC7A11, and thus increases the import of cysteine for GSH synthesis (Ogiwara et al., 2019). These studies provide a novel perspective into the association between epigenetic regulation and ROS production, which may further improve targeting of ferroptosis for oncotherapy.

Other participants involved in ferroptosis. The RAS family members (HRAS, NRAS and KRAS) are frequently mutated in several types of cancer and potentially activate oncogenic pathways to promote tumorigenesis. Small molecules that are specifically lethal to cells bearing oncogenic mutant RAS expression have been successfully identified (Torrance et al., 2001; Shaw et al., 2011; Weïwer et al., 2012). Two oncogenic RAS selective lethal (RSL) molecules termed RSL3 and erastin were first isolated in the 2000s, and were later demonstrated to trigger ferroptosis (Dolma et al., 2003; Yang and Stockwell, 2008). However, induction of ferroptosis proceeded in a both Ras-dependent and independent manner, whereas inhibition of the RAS/RAF/MEK/ERK (all of which are members of the MAPK family), results in inhibition of ferroptosis in cancer cells with Ras mutations (Yagoda et al., 2007). However, associations between Ras and ferroptosis signaling remain contested, and thus require further study.

## Ferroptosis in cancer Therapy

Implications of ferroptosis inducers in cancer. Plasticity of the cell state enables cancer cells to become resistant to various types of cancer therapies; however, the adapted genetic alterations could unexpectedly and concurrently increase the cancer cells susceptibility to other forms of death, or even induce these forms of cell death more directly. Cancer cells with a mesenchymal phenotype are typically associated with property of metastasis and resistance to cancer therapies, mostly incorporating classical apoptosis-induced manners, across different cancer lineages, and this cell state is specifically characterized by elevated synthesis of polyunsaturated lipids, which, coincidentally, are acquiescent substrates for lipid peroxidation during ferroptosis and therefore converges a dependency on GPX4. Featuring with high mesenchymal phenotypes, cancer cells of melanoma, prostate cancer and sarcomas, which are respectively resistant to the original cancer therapy, show upregulated susceptibility to GPX4-targeted ferroptosis (Hangauer et al., 2017; Viswanathan et al., 2017). These studies demonstrate that cells with significant resistance to other forms of RCD may in fact become selectively more sensitive to ferroptosis. Considering the frequent mutations of SLC7A11 in several types of cancer, triggering ferroptosis for cancer therapy may be a promising approach (Koppula et al., 2018; Combs and DeNicola, 2019).

System X_c_^−^ is essential for the transport of small molecule nutrients required for maintenance of cell proliferation and survival, which in turn makes system X_c_^−^ a promising target in oncotherapy (Lu et al., 2017). Erastin, which directly inhibits system X_c_^−^, is the most commonly used approach for induction of ferroptosis; however, the poor water solubility and unstable metabolic properties of erastin substantially impair its use clinically (Yang et al., 2014). Efforts have been made to overcome this difficulty; in triple negative breast cancer (TNBC) cells, erastin was encapsulated into exosomes and covered with folate, as folate receptors are extensively present throughout TNBC cells, and using this modified erastin complex, ferroptosis was effectively induced *in vivo* (Yu et al., 2019b). Piperazine erastin, erastin with a piperazine moiety attached to the aniline ring, was shown to possess improved solubility and stability compared with erastin, and displayed improved anti-tumor activity in a xenograft model established using HT-1080 cells (Dixon et al., 2012; Yang et al., 2014). Similarly, an Food and Drug Administration approved drug, sulfasalazine, which is normally used as an anti-inflammatory drug, can inhibit system X_c_^−^ and trigger ferroptosis in a range of cancer cell lines (Yang et al., 2014; Yu et al., 2019a), whereas sorafenib, a multi-kinase inhibitor, efficaciously induced ferroptotic cell death in hepatocellular carcinoma, and this was pharmacologically rescued by ferropstatin-1, and genetically suppressed by retinoblastoma protein overexpression (Louandre et al., 2013; Louandre et al., 2015).

Synthesizing cysteine from methionine, which bypasses system X_c_^−^, enables cells to evade ferroptosis induced by inhibition of system X_c_^−^ (Stockwell et al., 2017). Targeting the key regulator - GPX4, has thus become an alternate option to trigger ferroptosis (Gao et al., 2016). RSL3 can target enzymes, such as cysteine and selenocysteine, and directly inactivates GPX4 by alkylating selenocysteine (Yang et al., 2014). Conversely, overexpression of GPX4 counteracts RSL-3 induced ferroptosis in several different cancer cell lines. In addition to RSL3, the class 2 FINs, consisting of other synthetic small molecules, such as ML162 and ML210, impair GPX4 enzymatic activity without attenuating GSH synthesis. The class 1 FINs, such as DPI2 and DPI10, elicit similar mechanisms to that of erastin to functionally prevent GPX4 activity via GSH depletion (Yang et al., 2014; Lei et al., 2020).

**FIGURE 2:**
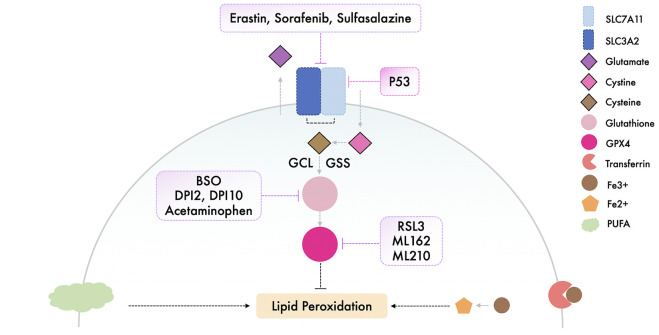
Illustration of the available ferroptosis inducers.

Triggering ferroptosis has shown to be an efficient method both *in vivo* and *in vitro* as a method of targeting and killing cancer cells, and these potential inducers should be studied further with regard to their suitability in a clinical setting (Florean et al., 2019; Hassannia et al., 2019). The currently available inducers of ferroptosis are illustrated and summarized respectively in Figure 2 and Table 1.

**TABLE 1:** Summary of the available ferroptosis inducers.

Compounds	Targets	Applied Cells/Animals	Reference
Erastin	VDAC2/3 and System X_c_^-^	BJeLR, HT1080, Calu-1,A-673, Hela, Jurkat T cells, U2PS, DU-145	Chan and Kwong (2000); Yang et al. (2005); Harada et al. (2011); Tomiotto-Pellissier et al. (2018); Dodson et al. (2019)
Piperazine Erastin (PE)	VDAC2/3 and System X_c_^-^	BJeLR, Nude mice	Yang et al. (2005)
Sorafenib	System X_c_^-^	HT1080, Calu-1, DU-145, Huh7, ACHN cells, Nude mice	Chan and Kwong (2000); Kastenhuber and Lowe (2017); Wu et al. (2019); Takahashi et al. (2020)
Sulfasalazine	System X_c_^-^	BJeLR, HT1080, Calu-1, DU-145, MDA-MB-231, BT54, MCF7, T47D	Dodson et al. (2019); Chan and Kwong (2000); Toyokuni et al. (2017)
Buthioninesulfoximine (BSO)	GSH	BJeLR, HTC116, A549	Yang et al. (2005); Chan and Kwong (2000)
Acetaminophen	GSH	HepG2, mouse hepatocytes	Lőrincz et al. (2015); Ishii et al. (2000)
FINs I (DPI2, DPI10)	GSH	BJeLR, HT1080	Yang et al. (2014)
RSL3	GPX4	BJeLR, HT1080, A549, Calu-1, HCT116, MIA PaCa-2, KBM7	Kwak et al. (2002); Sasaki et al. (2002)
FINs II (ML162, ML210)	GPX4	BjeLR, A549	Yang et al. (2014); Lei et al. (2020)

Immunotherapy and ferroptosis. Immunogenic apoptosis (IA), which is characterized by the release of damage-associated molecular patterns, such as ATP and chromatin-binding protein high-mobility group B1 (HMGB1) (Apetoh et al., 2007; Michaud et al., 2011; Garg et al., 2012), underline the importance of immune response activation in triggering death of malignant cancer cells (Zhang et al., 2019). Interestingly, it was found that HMGB1 is released during ferroptosis, which is respectively indued by erastin, RSL3, sorafenib and FIN56, in an autophagy-dependent manner (Dixon et al., 2012). This discovery consequently addresses the hypothesis that induction of ferroptosis in cancer cells can potentially trigger the innate immune response. Immunotherapy-activated CD8^+^ T cells have been shown to facilitate ferroptosis by enhancing lipid peroxidation in cancer cells, which in-turn increases the anti-tumor efficiency of immunotherapy. The IFNγ secreted by CD8^+^ T cells downregulates the expression of SLC7A11 and SLC3A2, and therefore attenuates the uptake of cystine by tumor cells, consequently enhancing lipid peroxidation in tumor cells, ultimately increasing ferroptosis (Dolma et al., 2003; Wang et al., 2019). With regard to this mechanism, increasing IFNγ levels in the tumor microenvironment appears to be a promising approach to remodel susceptibility of cancer cells to ferroptosis. Combined use of checkpoint immunotherapy and radiotherapy significantly increases Ki67^+^CD8^+^ T cell infiltration and concurrently increases expression of the Ataxia-Telangiectasia mutated gene. As a result, IFNγ is abundantly secreted in to the tumor microenvironment, which further promotes ferroptosis (Lang et al., 2019). In addition to apoptosis, ferroptosis provides a novel mechanism by which CD8^+^ T cells mediate tumor elimination *in vivo*. These studies suggest that targeting the mechanisms underlying ferroptosis may improve the efficiency of cancer immunotherapy.

## Conclusion

Ferroptosis is a more recently discovered form of RCD, that has been implicated as a target for cancer due to its pharmacologically/genetically accessible characteristics. Significant progress has been made to better exploit ferroptosis, including the development of inducers of death of cancer cells; however, notable challenges remain to be overcome. The machinery underlying ferroptosis is not fully understood; however, targeting the key modulators of this unique signaling method has provided promising efficacy in inducing/inhibiting ferroptotic death. High intracellular levels of redox-active iron (Fe^2+^) is a prerequisite for induction of ferroptosis. In fact, hydroxyl radicals generated by the Fenton reaction are the primary source of ROS facilitating ferroptotic lipid peroxidation. Esterification of carbohydrate based energy sources, such as PUFAs, is essential for the synthesis of lipid peroxidation substrates, which include AA-PE, Ada-PE and the more recently identified PUFA-ePLs. However, the antioxidant capacity of cells is regulated by GSH-GPX4 and NADH-FSP1-CoQ_10_ signaling axis, which efficiently counteracts intracellular lipid peroxidation and thereby prevents ferroptosis. Moreover, P53 and NRF2 are also shown to comprehensively interact with ferroptosis signaling, which consequently exploits candidate targets for management of ferroptotic cascade. Targeting the biological processes described above indeed trigger/attenuate ferroptosis; however, plasticity and heterogeneity of cancer cells enables them to potentially become resistant to ferroptosis. Thus, developing a means of increasing ferroptosis susceptibility alongside the use of ferroptosis inducers may improve the efficacy of the inducers. Interestingly, ferroptosis is found to actively participate in cancer immunotherapy, providing a novel perspective for treatment of cancer by combining the therapeutic approaches for regulating ferroptosis with immunotherapy-based approaches. Moreover, associations between chromatin accessibility and ROS production may also provide alternative methods for exploitation of ferroptosis clinically. Nevertheless, further studies are required to assess, develop and test these methods.

As the understanding of ferroptosis and its association with several other biological processes increases, targeting ferroptosis in cancer treatment may prove to be a clinically viable method, either alone or as an adjuvant. Studies on ferroptosis in oncotherapy are thus encouraged, and it is considered that an interdisciplinary approach may improve the impact of the studies, and thus the clinical value of the outcomes.
